# Changes in peripheral blood cytokines in patients with severe fever with thrombocytopenia syndrome

**DOI:** 10.1002/jmv.26877

**Published:** 2021-03-11

**Authors:** Zhiquan He, Bohao Wang, Yi Li, Kai Hu, Zhijie Yi, Hongxia Ma, Xingle Li, Wanshen Guo, Bianli Xu, Xueyong Huang

**Affiliations:** ^1^ Department of Infectious Disease Control and Prevention Henan Province Center for Disease Control and Prevention Zhengzhou China; ^2^ College of Public Health, Zhengzhou University Zhengzhou China; ^3^ Henan Key Laboratory of Pathogenic Microorganisms, Henan Province Center for Disease Control and Prevention Zhengzhou China; ^4^ Health Policy Research Center Henan Academy of Medical Sciences Zhengzhou China

**Keywords:** asymptomatic infected persons, cytokines, patients, severe fever with thrombocytopenia syndrome, viral load

## Abstract

Severe fever with thrombocytopenia syndrome (SFTS) is recognized as an emerging infectious disease. This study aimed to investigate the pathogenic mechanism of SFTS. A total of 100 subjects were randomly included in the study. Cytokine levels were detected by enzyme‐linked immunosorbent assay and the viral load was detected by micro drop digital PCR. The results showed that levels of interleukin‐6 (IL‐6), IL‐8, IL‐10, IFN‐inducible protein‐10 (IP‐10), monocyte chemoattractant protein‐1 (MCP‐1), macrophage inflammatory protein‐1α (MIP‐1α), transforming growth factor‐β1 (TGF‐β1), and regulated upon activation normal T cell expressed and secreted factor (RANTES) differed significantly among the SFTS patient group, healthy people group, and asymptomatic infection group (*p* < .05). Compared to the healthy people group, the patient group had increased cytokine levels (IL‐6, IL‐10, IP‐10, MCP‐1, and IFN‐γ) but reduced levels of IL‐8, TGF‐β1, and RANTES (*p* < .0167). IL‐6, IL‐8, IL‐10, IP‐10, MCP‐1, MIP‐1α, TGF‐β1, and the RANTES levels had different trends after the onset of the disease. IL‐6, IL‐10, IP‐10, and MCP‐1 levels in severe patients were higher than those in mild patients (*p* < .05). There was a positive correlation between viral load and IL‐6 and IP‐10 but a negative correlation between viral load and RANTES. SFTSV could cause a cytokine change: the cytokine levels of patients had different degrees of fluctuation after the onset of the disease. The levels of IL‐6 and IL‐8 in the asymptomatic infection group were found between the SFTS patients group and the healthy people group. The levels of IL‐6, IL‐10, IP‐10, and MCP‐1 in the serum could reflect the severity of the disease, and the levels of IL‐6, IP‐10, and RANTES were correlated with the viral load.

## 1 INTRODUCTION

Severe fever with thrombocytopenia syndrome (SFTS) is a novel emerging infectious disease caused by the SFTS virus (SFTSV), a tick‐borne phlebovirus of the family *Phenuiviridae* in the order *Bunyavirales*. However, according to the nomenclature by the International Committee of Taxonomy of Viruses (ICTV), SFTSV has been classified into the Genus *Banyangvirus*, Family *Phenuiviridae*, and re‐named as the Huaiyangshan banyangvirus. In this article, we have referred to it as “SFTSV”.^1^, ^2^ The clinical symptoms of SFTS patients are characterized by fever, thrombocytopenia, leukopenia, gastrointestinal and neurological symptoms, as well as abnormal concentrations of laboratory parameters.^3^, ^4^ As the case‐fatality rate of SFTS ranges from 5% to over 40%, with 0.3–11.3% seroprevalence of anti‐SFTSV antibodies in healthy individuals living in endemic areas (China, Japan, Korea) and no effective therapies or vaccines, understanding the pathogenic mechanism of SFTS is important for this disease.^5^, ^6^, ^7^, ^8^

Cytokines are small molecular polypeptides with immunological activity synthesized and released by activated lymphocytes or some tissue cells. Complications or, ultimately, death arising from virus infections are often associated with hyperinduction of pro‐inflammatory cytokine production, which is also known as a “cytokine storm”.^9^ The outcomes in SFTS patients include multiple organ dysfunction syndrome (MODS), shock, disseminated intravascular coagulation, and death.^10^, ^11^ Cytokine storm has been confirmed to be associated with MODS, and the correlation of cytokine levels between SFTS patients relative to a healthy population was reported in previous studies.^12^, ^13^, ^14^, ^15^

In this study, the serum cytokine profile of patients with SFTS infection with symptomatic disease, those with SFTS antibodies, but no history of clinical illness, and healthy individuals were examined to explore whether there are differences in serum cytokine. The correlation between cytokine levels and viral load was measured for the serum samples to investigate the patterns of changes and clinical significance.

## 2 MATERIALS AND METHODS

### 2.1 Study design

From May 2018 to July 2019, 131 confirmed SFTS patients admitted to a hospital in Xinyang, Henan, China were retrospectively summarized. Based on the integrity of the collected data, a total of 100 patients were included in the study. At the same time, 100 healthy people and 100 asymptomatic infected persons were randomly selected from the epidemiological survey on SFTSV infection status of the natural population in Xinyang city from 2016 to 2018 by the Henan Center for Disease Control and Prevention (CDC). The gender, age, occupation, and marital status of the healthy people group and asymptomatic infection group were matched with those of the patient group.

### 2.2 Inclusion criteria

SFTS patients: epidemiological history, clinical signs, and laboratory tests were diagnosed; patients met one or more of the following criteria: (1) the virus was isolated from the patient's samples; (2) SFTSV RNA was detected in the patient's serum; (3) a fourfold or greater increase in antibody titers was detected between paired patient serum samples collected from the acute and convalescent phases of infection.^16^

Mild SFTS cases: Low fever (37.2–39°C), mild fatigue, and gastrointestinal symptoms, the platelet count was (50–130) × 10^9^/L, and the levels of aspartate aminotransferase (AST), alanine aminotransferase (ALT), creatine kinase (CK), and lactate dehydrogenase (LDH) were less than two times the upper limit of normal (ULN) values.

Severe SFTS cases: high fever (39–40°C), fatigue, obvious gastrointestinal symptoms, neurological symptoms, the platelet count was (30–50) × 10^9^/L, and sharply elevated (more than five times ULN) of LDH, ALT, AST, and CK.^17^

Healthy people: according to the epidemiological history, clinical manifestations, and laboratory test results, the diagnosis should meet one or two of the following criteria: (1) laboratory tests showed that SFTSV nucleic acid; (2) SFTSV immunoglobulin G (IgG) or IgM antibodies were negative in serum samples.

Asymptomatic infected persons: there were no clinical symptoms (such as fever, cough, and sore throat) and the serum samples were positive for SFTSV or serum specific IgM antibody.

### 2.3 Sample and data collection

Serum samples coated with EDTA were collected from patients on admission during the acute phase of SFTSV infection (within 7 days of onset) in the 990 Hospital. After that, serum samples were collected every other day until discharge. Patients were divided into mild and severe groups based on clinical symptoms and progressions. Serum samples of asymptomatic infected persons and healthy people were collected during the epidemiological survey. All serum samples were frozen immediately after collection in the primary laboratory and were transported to the Henan CDC the next day. The serum samples were stored at −80°C until laboratory analysis. A structured questionnaire was used to collect sociodemographic and clinical data from the subjects.

### 2.4 Enzyme‐linked immunosorbent assay (ELISA) test cytokine levels and antibodies (IgG and IgM)

The cytokine levels were detected in serum samples using ELISA kits (MultiSciences Biotech Co., Ltd.) according to the manufacturer's protocol. Cytokines, including interleukin‐2 (IL‐2), IL‐6, IL‐8, IL‐10, interferon‐γ (IFN‐γ), IFN‐inducible protein‐10 (IP‐10), monocyte chemoattractant protein‐1 (MCP‐1), macrophage inflammatory protein‐1 (MIP‐1), transforming growth factor‐β1 (TGF‐β1), tumor necrosis factor‐α (TNF‐α), and regulated upon activation normal T cell expressed and secreted factor (RANTES) were tested. For this ELISA kit, 50 µl of serum samples, twofold diluted standard, and diluted detection antibody were added. Samples were incubated at room temperature for 2 h on a microplate shaker set at 300 revolutions per minute (rpm) and washed with washing buffer, and 100 µl of diluted streptavidin‐horseradish peroxidase (HRP) was added. Plates were developed by using a substrate solution after samples were incubated and washed. The detection of antibodies (IgG and IgM) to SFTSV is previously described.^18^

### 2.5 Detection of viral load by reverse‐transcriptase droplet digital polymerase chain reaction (RT‐ddPCR)

One‐step RT‐ddPCR was performed in a 20 µl reaction volume containing 4 µl of RNA sample, 5 µl of supermix for probes, 2 µl of reverse transcriptase, 0.5 µl of probes, 1.6 µl of target primers, 1 µl of DTT, and 4.3 µl of RNase‐free water. The 20‐µl reaction volume and 70 µl droplet generation oil for probes were, respectively, loaded into the sample wells and oil wells of a disposable droplet generator cartridge. Then, droplets were generated by a QX200 droplet generator device, and 40 µl of mixed liquid was carefully transferred to a 96‐well PCR plate (Bio‐Rad). One‐step RT‐ddPCR amplification was performed with the following conditions: 48°C for 60 min, 95°C for 10 min, 40 cycles of 95°C for 30 s, and 58°C for 1 min followed by 98°C for 10 min. Additionally, no template control was included in this assay.^19^

### 2.6 Statistical analysis

Statistical analyses were performed using SPSS 21.0 software (SPSS, Inc.). Means and standard deviations (*SD*s) were described as continuous variables with normal distribution; medians and interquartile (IQ) ranges were used for abnormal distribution. The Shapiro‐Wilk test was conducted to test the normal distribution. If data met the criteria for the abnormal distribution, then a nonparametric test was performed to evaluate the differences in cytokine levels. Otherwise, the analysis of variance was used. The categorical variables were calculated with the *χ* ^2^ test. The correlations between variables were assessed using the Pearson test. A *p* value of < .05 was considered to be significant. Besides this, when we used the Bonferroni method for multiple comparisons, the adjusted α value was 0.0167, then *p* < .0167 was considered to be statistically significant.^20^

### 2.7 Ethics approval and consent to participate

The study was approved by the Ethical Committee of Henan CDC, and the committee's reference number is 2016‐KY‐002‐02. All participants provided written informed consent for use of their samples in research.

## 3 RESULTS

### 3.1 Respondent characteristics

For the SFTS patient group, the mean age was 65.45 ± 9.22 years, and 50 were female. The mean age of the healthy people group was 66.57 ± 10.18 years, with 44 males and 56 females. For the asymptomatic infected person group, the mean age was 64.24 ± 8.98 years, and 39 were males. The results of the analysis of variance showed that there were no significant differences in age, gender, occupation, and marital status among the SFTS patients' group, healthy people group, and asymptomatic infection group (*p* > .05) (Table 1).

**Table 1:** Comparison of research subjects in  general

Characteristics	SFTS patient group (*n* = 100)	Healthy people group (*n* = 100)	Asymptomatic infected person group (*n* = 100)	*χ*^2^/*F*	*p* value
Age, year (mean ± *SD*)	65.45 ± 9.22	66.57 ± 10.18	64.24 ± 8.98	1.510	.222
Sex, *n* (%)				2.458	.293
Male	50 (50)	44 (44)	39 (39)		
Female	50 (50)	56 (56)	61 (61)		
Marital status, *n* (%)				1.367	.505
Married	92 (97)	87 (87)	90 (90)		
Unmarried/divorced/widowed	8 (3)	13 (13)	10 (10)		
Occupation, *n* (%)				2.589	.274
Farmer	89 (89)	93 (93)	86 (86)		
Others	11 (11)	7 (7)	14 (14)		

### 3.2 Cytokine levels in serum

The levels of 11 cytokines were quantitatively determined in serum samples of each respondent on admission. We analyzed cytokine levels among the three groups by the nonparametric Kruskal‐Wallis *H* test. The results showed that median IL‐6 levels were 71.39 pg/ml (IQ, 33.35–141.60 pg/ml), 25.32 pg/ml (IQ, 12.40–29.32 pg/ml), and 29.80 pg/ml (IQ, 17.20–39.56 pg/ml) in the SFTS patient, healthy people and asymptomatic infected person groups, respectively, and showed significant differences (*p* < .001). Levels of IL‐8, IL‐10, IP‐10, MCP‐1, MIP‐1α, IFN‐γ, TGF‐β1, and RANTES differed significantly (*p* < .05), but there was no significant difference in TNF‐α levels among the three groups (*p* = .954); IL‐2 was not detected due to the lack of high sensitivity (Table 2). Multiple comparisons were performed to determine differences in the three groups. Serum IL‐6, IL‐8, MCP‐1, and TGF‐β1 levels were identified as significantly different by multiple comparisons among the three groups (*p* < .0167), but IL‐10, IP‐10, MIP‐1α, IFN‐γ, TNF‐α, and RANTES levels exhibited no significant differences (*p* > .0167).

**Table 2:** Characteristics of subjects and cytokine levels included in the study

Characteristics	SFTS patient group (*n* = 100)^a^	Healthy people group (*n* = 100)	Asymptomatic infected person group (*n* = 100)	*p* value^b^	*P* value^c^	*p* value^d^	*p* value^e^
IL‐6	71.39	25.32	29.80	<.001*	<.001*	.005*	<.001*
	(33.35–141.60)^f^	(12.40–29.32)	(17.20–39.56)				
IL‐8	66.28	70.50	69.27	<.001*	<.001*	.010*	<.001*
	(66.02–66.85)	(68.03–76.85)	(66.49–74.21)				
IL‐10	20.29	5.19	5.56	<.001*	<.001*	.096	<.001*
	(11.09–42.03)	(3.29–6.54)	(4.29–7.19)				
IP‐10	1124.53	58.05	61.53	<.001*	<.001*	.327	<.001*
	(444.24–2173.64)	(41.99–75.50)	(45.91–79.04)				
MCP‐1	195.40	86.83	54.22	<.001*	<.001*	.004*	<.001*
	(80.68–405.09)	(52.18–126.95)	(35.44–103.75)				
MIP‐1α	684.80	684.16	683.28	.157	.001*	.010*	.0011*
	(682.80–711.08)	(682.85–689.99)	(681.93–686.22)				
IFN‐γ^g^	36.86 (18.38–83.93)	10.36 (3.81–42.01)	6.06 (3.91–12.72)	.003*	<.001*	.665	<.001*
	(*n* = 73)	(*n* = 25)	(*n* = 23)				
TGF‐β1	980.25	3654.05	4694.88	<.001*	<.001*	<.001*	<.001*
	(430.62–1609.65)	(2915.33–4268.67)	(3597.08–5083.66)				
		
TNF‐α^g^	73.78 (27.27–203.28)	85.72 (40.34–104.05)	56.95 (36.54–187.91)	.726	.901	.767	.954
	(*n* = 20)	(*n* = 13)	(*n* = 11)				
RANTES	1236.15	1671.83	2011.91	<.001*	<.001*	.033	<.001*
	(787.60–1636.08)	(1330.82–2089.53)	(1300.61–2630.62)				

Abbreviations: IFN‐γ, interferon‐γ; IL‐6, interleukin‐6; IP‐10, IFN‐inducible protein‐10; MCP‐1, monocyte chemoattractant protein‐1; MIP‐1α, macrophage inflammatory protein‐1α; RANTES, regulated upon activation normal T cell expressed and secreted factor; TNF‐α, tumor necrosis factor‐α; TGF‐β1, transforming growth factor‐β1.

*Note*: Unit: pg/ml.

^a^ The cytokine levels were measured in SFTS patients on admission.

^b^ The SFTS patient group compared with the healthy people group.

^c^ The SFTS patient group compared with the asymptomatic infected person group.

^d^ The healthy people group compared with the asymptomatic infected person group.

^e^ Comparison among the three groups.

^f^ Denotes the median (IQ).

^g^ Denotes there was a lack of data.

* Denotes had statistical differences.

For the SFTS patient group, IL‐6, IL‐10, IP‐10, MCP‐1, and IFN‐γ levels were significantly increased, but levels of IL‐8, TGF‐β1, and RANTES were decreased compared to those in both the asymptomatic infected person and healthy people groups (*p* < .0167). MIP‐1α levels in the patient group were higher than those in the asymptomatic infected person group; however, there was no significant difference in the healthy people group. Notably, serum TNF‐α levels showed no difference compared to the control and asymptomatic infected person groups (*p* > .0167).

For the healthy people group, IL‐10, IP‐10, MIP‐1α, IFN‐γ, TNF‐α, and RANTES levels exhibited no significant differences compared to the asymptomatic infected person group, but IL‐6, IL‐8, MCP‐1, and TGF‐β1 levels were significantly different. Serum IL‐8 levels were the highest, and IL‐6 levels were the lowest in this group. Compared to the asymptomatic infected group, MCP‐1 levels were higher than the control group. Otherwise, TGF‐β1 levels were lower than those in the recessive‐infected person group (*p* < .0167).

**Figure 1:**
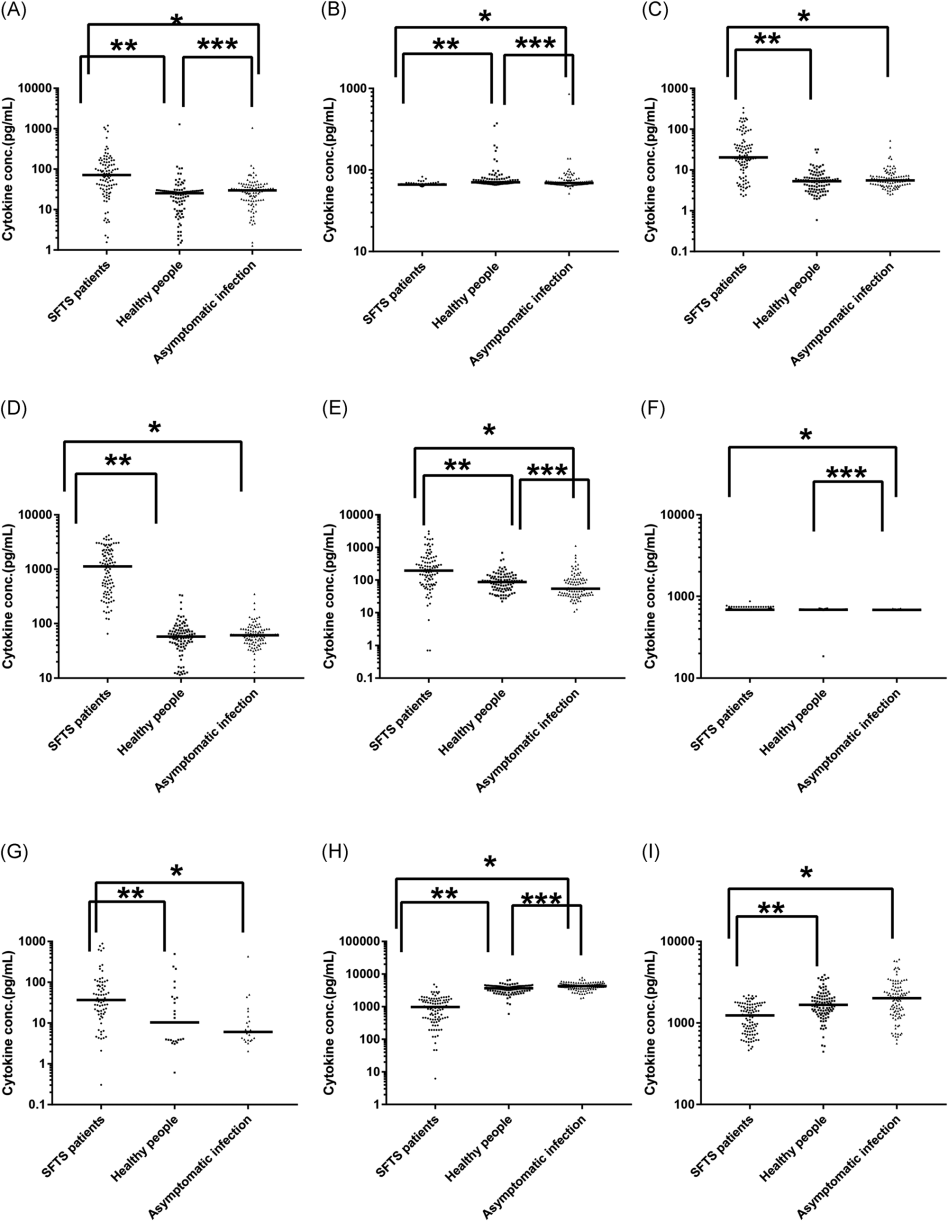
Comparison of cytokine levels among the SFTS patient on admission, healthy people group, and asymptomatic infected person groups. Unit: pg/ml. A, IL‐6, (B) IL‐8, (C) IL‐10, (D) IP‐10, (E) MCP‐1, (F) MIP‐1α, (G) IFN‐γ, (H)TGF‐β1, and (I) RANTES. Horizontal bars indicate the group median. *p < .0167 between the patient and asymptomatic infected person groups. **p < .0167 between the healthy people and patient groups, ***p < .0167 between the healthy people and asymptomatic infected person groups. IFN‐γ, interferon‐γ; IL‐6,  interleukin‐6; IP‐10, IFN‐inducible protein‐10; MCP‐1, monocyte chemoattractant protein‐1; MIP‐1α, macrophage inflammatory protein‐1α; RANTES, regulated upon activation normal T cell expressed and secreted factor; SFTS,  thrombocytopenia syndrome; TGF‐β1, transforming growth factor‐β1

For the asymptomatic infected person group, serum TGF‐β1 levels were the highest among the three groups. RANTES levels were increased in this group compared to the patient group but exhibited no significant difference in the healthy people group (*p* > .0167) (Figure 1).

### 3.3 Dynamic changes of serum cytokines in patients

**Figure 2:**
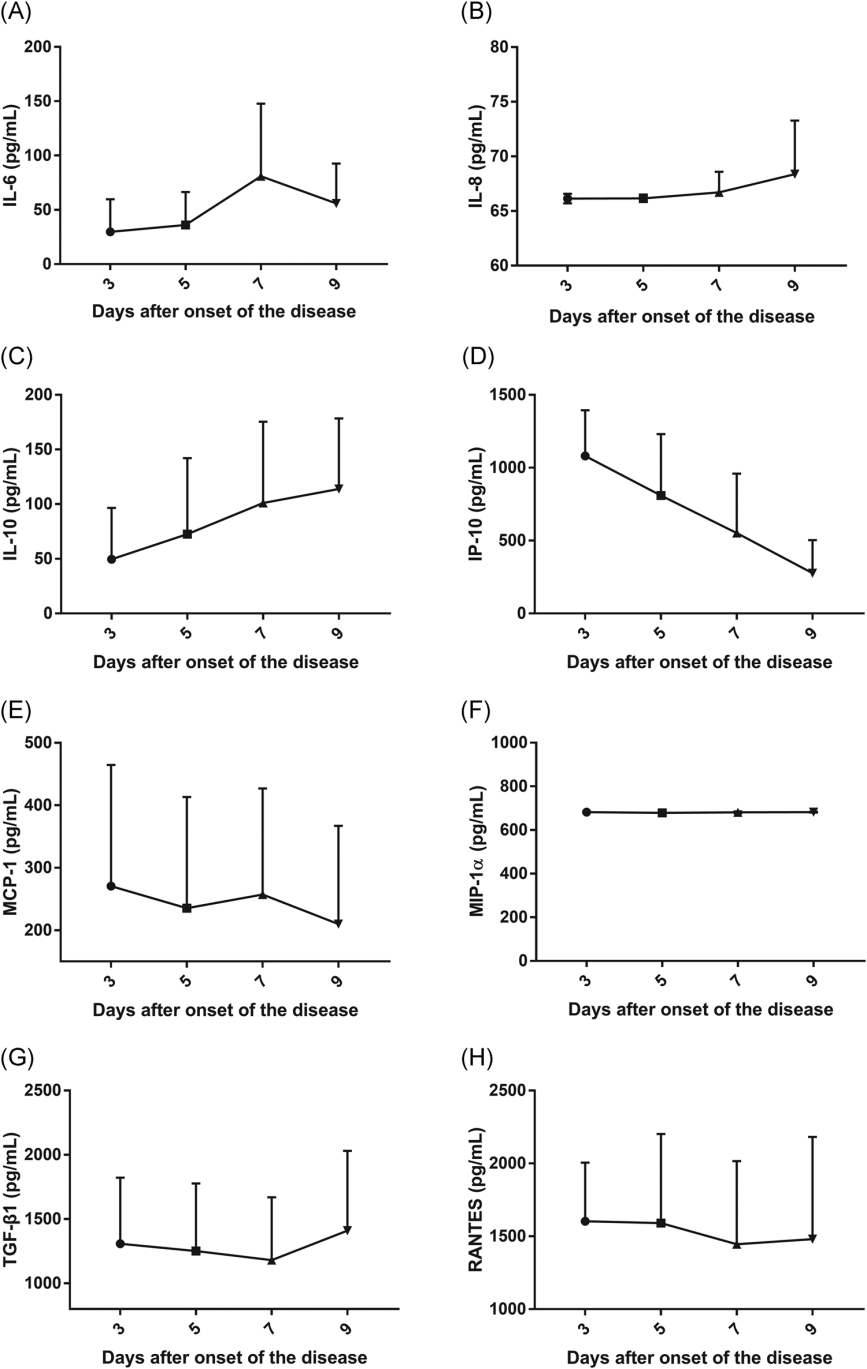
Dynamic changes of serum cytokines in 22 mild SFTS patients after onset of the disease

The cytokine levels in serum samples of 22 mild patients were measured during hospitalization at Days 3, 5, 7, and 9 due to most patients dropping below the measurable level after the tenth day. IL‐8 and IL‐10 gradually increased during our observation period, but IP‐10 gradually decreased. MIP‐1α did not significantly fluctuate after hospitalization. IL‐6, MCP‐1, TGF‐β1, and RANTES had different trends, either increasing first and then decreasing or vice versa. IFN‐γ and TNF‐α were not described due to incomplete data (Figure 2).

### 3.4 Comparison of cytokine levels in the SFTS patient group

As shown in (Table 3), 78 of 100 were patients with mild symptoms and 22 were patients with severe symptoms. The mean age of participants with mild symptoms was 65.44 ± 9.47 years, and that of patients with severe symptoms was 65.50 ± 8.49 years. Age and gender were not significantly different in patients with severe and mild symptoms (*p* > .05). Serum IL‐6, IL‐10, IP‐10, MCP‐1, and IFN‐γ levels were significantly increased in patients with severe symptoms relative to patients with mild symptoms (*p* < .05). However, the levels of IL‐8, MIP‐1α, TGF‐β1, TNF‐α, and RANTES were not significantly different between mild and severe patients (*p* > .05).

**Table 3:** Comparison of demographic and cytokine differences among SFTSV patients relative to the acuity of disease

Characteristics	Mild disease (*n* = 78)	Severe disease (*n* = 22)	χ^2^/*t*/*z*	*p* value
Age, year (mean ± SD)	65.44 ± 9.47	65.50 ± 8.49	0.029	.977
Sex (male)	41 (52.56%)	9 (40.91%)	0.932	.334
*n* (%)				
IL‐6	55.11	133.87	14.429	<.001
	(26.85–99.29)^a^	(94.95–204.53)		
IL‐8	66.26	66.48	1.367	.242
	(65.98–66.84)	(66.12–67.11)		
IL‐10	18.80	99.02	14.556	<.001
	(7.36–34.69)	(21.56–159.05)		
IP‐10	852.19	1944.40	10.968	.001
	(345.28–1701.48)	(1125.84–2453.77)		
MCP‐1	185.51	569.56	6.980	.008
	(76.79–230.37)	(105.40–1283.45)		
MIP‐1α	684.26	685.95	2.594	.107
	(682.09–710.71)	(683.95–742.47)		
IFN‐γ^b^	27.69(13.33–50.76)	103.08 (51.20–212.72)	19.032	<.001
	(*n* = 52)	(*n* = 21)		
TGF‐β1	1116.73	588.61	2.661	.103
	(442.4–1628.11)	(409.84–1242.41)		
TNF‐α^b^	44.21(21.93–178.94)	120.50 (48.08–338.22)	1.622	.203
	(*n* = 12)	(*n* = 8)		
RANTES	1260.07	1134.79	0.020	.680
	(785.09–1687.11)	(815.88–1540.5)		

*Note*: Unit, pg/ml.

Abbreviations: IFN‐γ, interferon‐γ; IL‐6, interleukin‐6; IP‐10, IFN‐inducible protein‐10; MCP‐1, monocyte chemoattractant protein‐1; MIP‐1α, macrophage inflammatory protein‐1α; RANTES, regulated upon activation normal T cell expressed and secreted factor; SFTSV, thrombocytopenia syndrome virus; TNF‐α, tumor necrosis factor‐α; TGF‐β1, transforming growth factor‐β1.

^a^ Denotes the median (IQ).

^b^ Denotes there was a lack of data.

### 3.5 Viral load in SFTS patients

**Figure 3:**
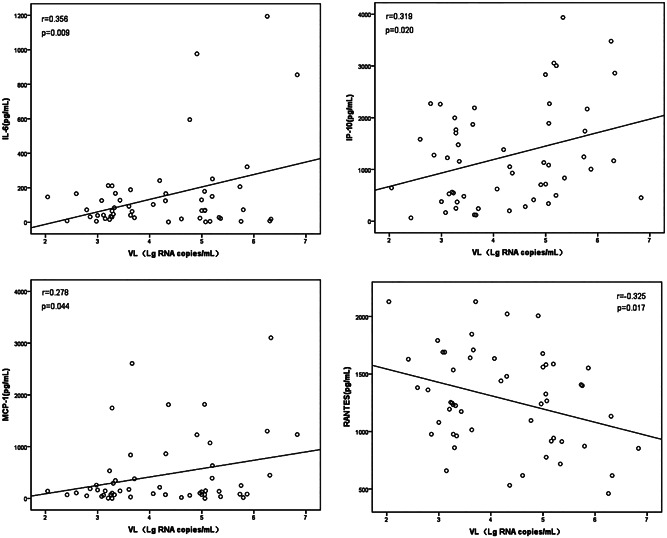
Correlation between viral load and cytokines. Correlations between variables were assessed using the Pearson test. *p* < .05 was considered significant. VL, viral load; Lg RNA copies/ml, Log_10_ RNA copies/ml

The SFTSV viral load in the blood of 53 patients on admission ranged from 0.11 × 10^3^ to 6.81 × 10^6^ copies/ml. The exponential average RNA viral load was 2.97 × 10^2^ copies/ml in 40 mild patients and 1.23 × 10^6^ copies/ml in 13 severe patients. The viral load was significantly higher in severe patients compared with mild patients (*p* < .05). Correlation analyses were conducted to investigate possible relationships between viral loads and the cytokines in SFTS patients during the acute phase of SFTSV infection. The results showed that positive correlations existed between viral load and IL‐6, IP‐10, and MCP‐1. A negative correlation existed between viral load and RANTES (Figure 3). The rest of the cytokines (IL‐8, IL‐10, MIP‐1α, IFN‐γ, TGF‐β1) and serum virus load showed no statistical correlations (*p* > .05).

## 4 DISCUSSION

To date, the pathogenesis of SFTSV infection has not been clearly defined. As we know, the immune response plays an important role in the prognosis of infectious diseases, especially viral infections.^21^ After consulting the relevant studies, we found that: disease severity of hemorrhagic fever with renal syndrome caused by hantavirus was correlated with some cytokines, including IL‐2, IL‐6, IL‐8, IL‐10, TGF‐β1, TNF‐α, and IP‐10^22^, ^23^; increased Crimean‐Congo hemorrhagic fever (CCHF) virus load and concentrations of IL‐6, IP‐10, MCP‐1, and TNF‐α were strongly associated with death in CCHF^24^, ^25^; and Rift Valley fever virus, a phlebovirus member, and cytokines were detected at significantly increased (IL‐8, MCP‐1, IP‐10, IL‐10) or decreased (RANTES) levels when comparing fatal cases to infected survivors and uninfected controls.^26^ Based on these previous studies, we analyzed eleven cytokines associated with SFTSV infection.

IL‐2 levels were not detected due to the very low content of IL‐2 in SFTS patients, levels of 7–8 pg/ml in healthy people, and lower than 1 pg/ml in SFTS patients.^14^ In the acute phase, both IL‐6 and IL‐10 were increased in the patient group compared to the healthy people group, possibly because IL‐6 is an important factor in inducing B cells to differentiate into plasma cells to produce antibodies upon virus infection, and IL‐10 has an anti‐inflammatory effect.^27^ ^,^ ^28^ The above results were similar to previously reported studies.^13^, ^14^, ^28^ Serum levels of IL‐8 were lower in the SFTSV‐infected patient group than the control group, possibly because IL‐8 functions as an immunological inflammatory regulatory factor in complex cytokine networks of body systems, the result was similar to that of Zhang et al.^29^, ^30^ After the onset of the disease, IL‐6 had a gradual increase between three and seven days, and IL‐8 and IL‐10 had the same trends between three and nine days. We inferred that levels of these cytokines saw dramatic changes in the infection process.

Except for RANTES and MIP‐1α, the other cytokines were increased in the patient group compared to the healthy people and asymptomatic infected person groups. The significant role of MCP‐1 and IP‐10 in virus‐infected disease progression has been reported.^31^ In this study, serum MCP‐1 and IP‐10 levels in the SFTS patient group were increased compared to the healthy people group. In addition to IP‐10, MCP‐1 and RANTES had large fluctuations after onset, suggesting that dynamic levels of cytokines in plasma might be closely related to the condition of this disease. Nevertheless, the limitation was that the severe patients in dynamic changes of serum cytokines were not observed due to the short time in hospital and fewer serum samples collected. IP‐10 and MCP‐1 had significant increases in severe patients compared with mild patients; these observations might be somewhat different from those of a previous study.^3^ The correlations between viral load and cytokines suggested that higher numbers of the virus were capable of inducing higher levels of IP‐10 and MCP‐1 while repressing the production of RANTES to a large extent.

IFN‐γ had a remarkable change among the three groups and was significantly elevated in patients with severe symptoms, the result was similar to that of the previous study.^21^ TNF‐α showed no significant differences among the three groups and the patients with severe and mild symptoms. However, some studies reported that high levels of TNF‐α were associated with the severity of the disease, and TNF‐α is a pro‐inflammatory cytokine known to have a key role in the pathogenesis of chronic immune‐mediated diseases.^32^, ^33^ Determining the reasons underlying this difference requires our in‐depth study and analysis.

## 5 CONCLUSION

SFTSV could cause a cytokine change: the cytokine levels of patients had different degrees of fluctuation after the onset of the disease, suggesting that the levels of cytokines might be changing dynamically in this process, rather than simply increasing or decreasing. The levels of serum cytokines (IL‐6, IL‐8) in the asymptomatic infection group were between the SFTS patients group and the healthy people group. The immune system of some infected people could clear SFTSV and they had no clinical symptoms. The levels of IL‐6, IL‐10, IP‐10, and MCP‐1 in serum could reflect the severity of the disease, and the levels of some cytokines (IL‐6, IP‐10, RANTES) were correlated with the viral load. Many patients were treated at basic hospitals, where viral load could not be detected due to the limitations of instruments and technology. IL‐6, IP‐10, and RANTES were tested by ELISA at basic hospitals to reflect viral load levels.

## CONFLICT OF INTERESTS

The authors declare that there are no conflict of interests.

## AUTHOR CONTRIBUTIONS

Conceptualization: Zhiquan He, Xueyong Huang; data curation: Zhiquan He, Bohao Wang, Yi Li; formal analysis: Zhiquan He, Zhijie Yi, Hongxia Ma; funding acquisition: Bianli Xu, Xueyong Huang; investigation: Zhiquan He, Yi Li, Zhijie Yi, Xueyong Huang; methodology: Zhiquan He, Bohao Wang, Kai Hu; project administration: Xueyong Huang; resources: Bianli Xu, Xueyong Huang; software: Zhiquan He, Bohao Wang, Xingle Li; validation: Bianli Xu, Xueyong Huang; visualization: Zhiquan He; writing—original draft: Zhiquan He, Bohao Wang; writing—review and editing: Wanshen Guo, Bianli Xu, Xueyong Huang. All authors read and approved the final manuscript.

### PEER REVIEW

The peer review history for this article is available at https://publons.com/publon/10.1002/jmv.26877

## Data Availability

The data that supports the findings of this study are available in the supplementary material of this article.
